# Mechanical and electrical properties of electrospun PVDF/MWCNT ultrafine fibers using rotating collector

**DOI:** 10.1186/1556-276X-9-522

**Published:** 2014-09-23

**Authors:** Shu-Hua Wang, Yong Wan, Bin Sun, Ling-Zhi Liu, Weijiang Xu

**Affiliations:** 1 College of Physics, Key Laboratory of Photonics Materials and Technology in Universities of Shandong, Qingdao University, Qingdao, 266071, People's Republic of China; 2 Départment d’Opto-Acousto-Electronique, IEMN, URM, CNRS 8520, Université de Valenciennes et du Hainaut Cambrésis, Valenciennes, Cedex, 59313, France

**Keywords:** Electrospinning, Multi-walled carbon nanotubes, Conductivity, Elasticity

## Abstract

Poly(vinylidene fluoride) (PVDF) ultrafine fibers with different proportions of multi-walled carbon nanotube (MWCNT) embedded have been fabricated using a modified electrospinning device with a rotating collector. With the increasing of MWCNT content, the *β* phase was noticeable enhanced, and the fibers became more elastic, which was manifested by Young's modulus decreased drastically. Furthermore, with adding the amounts of MWCNTs, the density of carbon nanotube (CNT)-CNT junctions among the fibers increased accordingly. When the MWCNT content was of 1.2 wt.%, a stable three-dimensional conducting network was formed. After this percolation threshold, the density of CNT-CNT junctions among the fibers tended to be a constant quantity, leading to a stabilized conductivity consequently. It is hoped that our results can be helpful for the fabrication of flexible devices, piezoelectric devices, force transducer, and so on.

**PACS:**

81.05.Qk; 81.16.-c

## Background

Electrospinning is a simple and versatile technique for fabricating ultrafine fibers with diameters ranging from several micrometers down to a few nanometers. With outstanding properties such as large surface area, high length/diameter ratio, flexible surface functionality, and tunable surface morphologies, the electrospun fibers have an underlying application in optoelectronics, sensors, catalysis, textiles, filters, fiber reinforcement, tissue engineering, drug delivery, wound healing, etc. [1-7]. During electrospinning process, when the electric field force reaches a certain threshold value, polymer droplet overcomes the surface tension and forms a jet trickle from the capillary Taylor cone vertex. After a series of vigorous whipping and/or splitting motion due to fluid instability and electrically driven bending instability, the products are deposited commonly as a nonwoven fibrous web on a collector. In order to improve the further application of the as-spun fibers, numerous researchers and groups have engaged in fabricating morphology-controlled electrospun micro/nanofibers, and it is delighted to notice that apart from fiber membranes without orientation, other fibrous structures and organization (e.g*.*, aligned fiber arrays, helical or wavy fibers, twisted fibrous yarns, patterned fibrous mats) based on not only polymers of synthetic or biological nature but also metals, metal oxides, ceramics, organic/organic, organic/inorganic, as well as inorganic/inorganic composite systems have been electrospun successfully via modified electrospinning process or collectors [8-10], which will extend further application of as-spun fibers in many fields.

As a semicrystalline polymer, poly(vinylidene fluoride) (PVDF) has aroused much attention due to its distinguished electroactive properties, nonlinear optical, strong corrosive, susceptibility, and high dielectric constant [11,12], which make it useful in a variety of fields such as sensors, actuators, and energy transducers [13]. PVDF consists of four different crystalline phases depending on the chain conformation of trans and gauche linkages: *α*, *β*, *γ*, and *δ*. Among these phases, the *α* phase is known as the most abundant form commercially available powders and films, and the *β* phase has the largest spontaneous polarization per unit cell and thus, exhibits the highest electroactive properties, responsible for most of PVDF's piezoelectric characters [14]. It is reported that electrospinning and blending PVDF with carbon nanotubes (CNTs) can increase the *β*-phase content in PVDF [15].

So far, the study of PVDF/CNT composites mainly focuses on the following three aspects: (1) the dielectric property of the composites and its CNT dispersion and loading dependence [16]; (2) enhancement of the *β*-phase crystal formation of PVDF in the doping of CNTs and the related property alterations [17,18]; (3) the electrical conductivity, its percolation behavior and other properties of the composites in the doping of CNTs [18-23]. Although numerous studies on PVDF/CNT nanofibrous composites have been published, new work in this field emerges consistently and continually. In the present work, well-aligned PVDF ultrafine fibers with different multi-walled carbon nanotube (MWCNT) contents (0.6%, 0.8%, 1%, 1.2%, 1.4%, 1.6%, 1.8%, and 2%) have been fabricated using a modified electrospinning device with a rotating collector. It is found that with the increasing content of MWCNTs from 0.6 to 2 wt.%, the *β* phase has been noticeable enhanced, and the composited fibers become much more elastic lying in the fact that Young's modulus decreased from 4.4 × 10^-2^ to 9.1 × 10^-3^ MPa. Moreover, with adding the amounts of MWCNTs, the density of CNT-CNT junctions among the fibers increased accordingly, forming a stable three-dimensional conducting network. After the three-dimensional network has been constructed (the percolation threshold) where MWCNT content was of 1.2 wt.%, the density of CNT-CNT junctions tend to be a constant quantity, resulting in a stabilized conductivity of the fibers.

## Methods

### Preparation of PVDF/MWCNT solution and electrospinning

**Figure 1:**
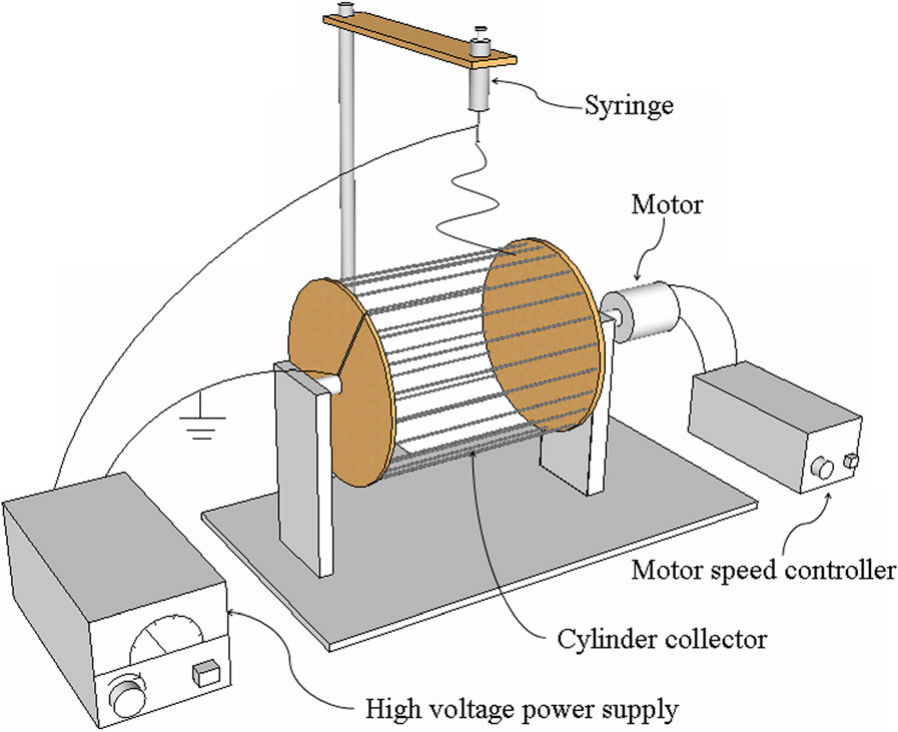
Schematic illustration of the electrospinning setup with a rotating wire-framed drum.

A mixture of dimethylformamide (DMF) and acetone (1:1 by weight) was used as the solvent. Subsequently, poly(vinylidene fluoride) (PVDF; average molecular weight of 270,000, Sigma-Aldrich, St. Louis, MO, USA) was dissolved in this solvent at a concentration of 18 wt.%, and under stirring for 2 h at 60°C. The MWCNTs, which were blended with carboxylic dispersing agent (trade name TNWDIS, Chengdu Organic Chemicals Co. Ltd., China) and acetone (1:8.4 by weight), were dispersed in the PVDF solution to make a final solution with MWCNT mass ratios of 0.6%, 0.8%, 1%, 1.2%, 1.4%, 1.6%, 1.8%, and 2%, respectively. However, it is relevant to emphasize that it was not possible to further increase the MWCNT proportion above 2 wt.% because of the low MWCNT dispersion.Herein, a rotating wire-framed drum was used as collector for fabricating aligned PVDF/MWCNT ultrafine fibers, as shown in Figure 1. The viscous fluid was loaded in a syringe with a stainless steel spinneret (inner diameter 0.72 mm), which was connected to the positive electrode of a high-voltage dc power supply (Tianjin Dongwen High Voltage Power Supply Co. Ltd., Tianjin, China) generating a constant voltage ranging from 0 to 30 kV. Here the applied voltage was of 16 kV. The negative electrode of the high-voltage dc power supply was attached to the rotating drum (height 4 cm, diameter 15.2 cm). The vertical distance (work distance) from the spinneret to the collector was about 15 cm. All experiments were carried out at room temperature, and the ambient humidity was from 20% to 70% relative humidity (RH).

### Characterization

To study the surface morphology and the size of electrospun fibers, a scanning electron microscope (SEM; JEOL JSM-6390, JEOL Ltd., Akishima-shi, Japan) and a transmission electron microscope (TEM; HITACHI H-9000, Hitachi, Ltd., Chiyoda-ku, Japan) were used. The crystalline phase or phases present in the composited fibers were identified by Fourier transform infrared (FTIR) spectroscopy using a Thermo Scientific Nicolet iN10 spectrometer (Thermo Fisher Scientific, Waltham, MA, USA), and absorbance data were processed for the wave number ranging from 600 to 1,600 cm^-1^. The X-ray diffraction patterns were recorded by a Bruker D8 Advance X-ray diffractometer (XRD; Bruker AXS, Inc., Madison, WI, USA). An electronic tensile testing machine (Jinan Hengrui machine Co. Ltd., Jinan, China) was used for the mechanical characterization of aligned electrospun fibrous membranes, and the electrical properties of the fibers were tested using a Keithley 6485 high-resistance meter system (Keithley Instruments, Inc., Cleveland, OH, USA).

## Results and discussion

### Morphologies

**Figure 2:**
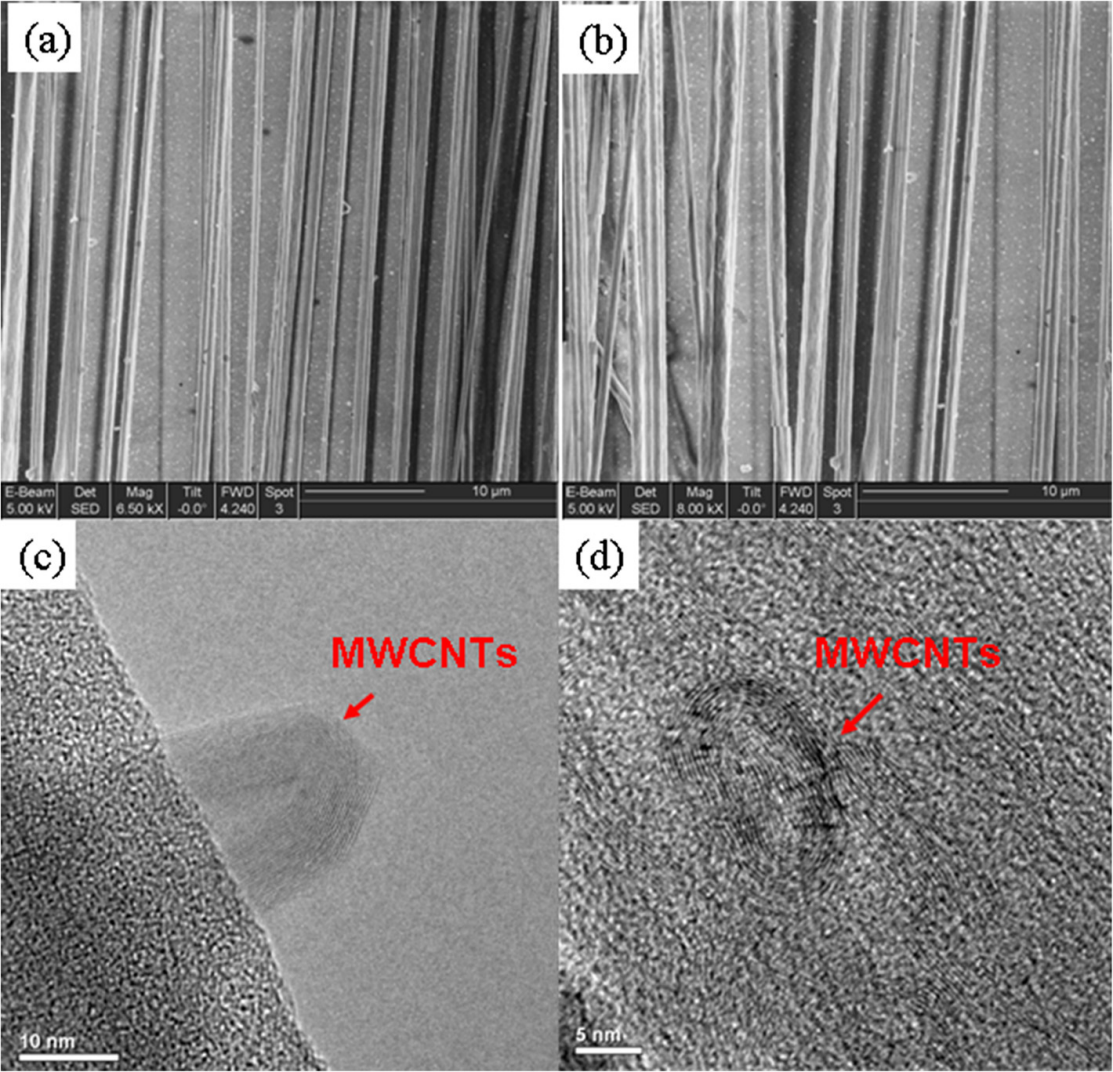
**SEM images of the aligned electrospun PVDF/MWCNTs ultrafine fibers collected by the rotating collector.** With MWCNT content of **(a)** 2 wt.% and **(b)** 0.6 wt.%. **(c,d)** TEM images demonstrate MWCNTs in the ultrafine fibers with curved conformation.

**Figure 3:**
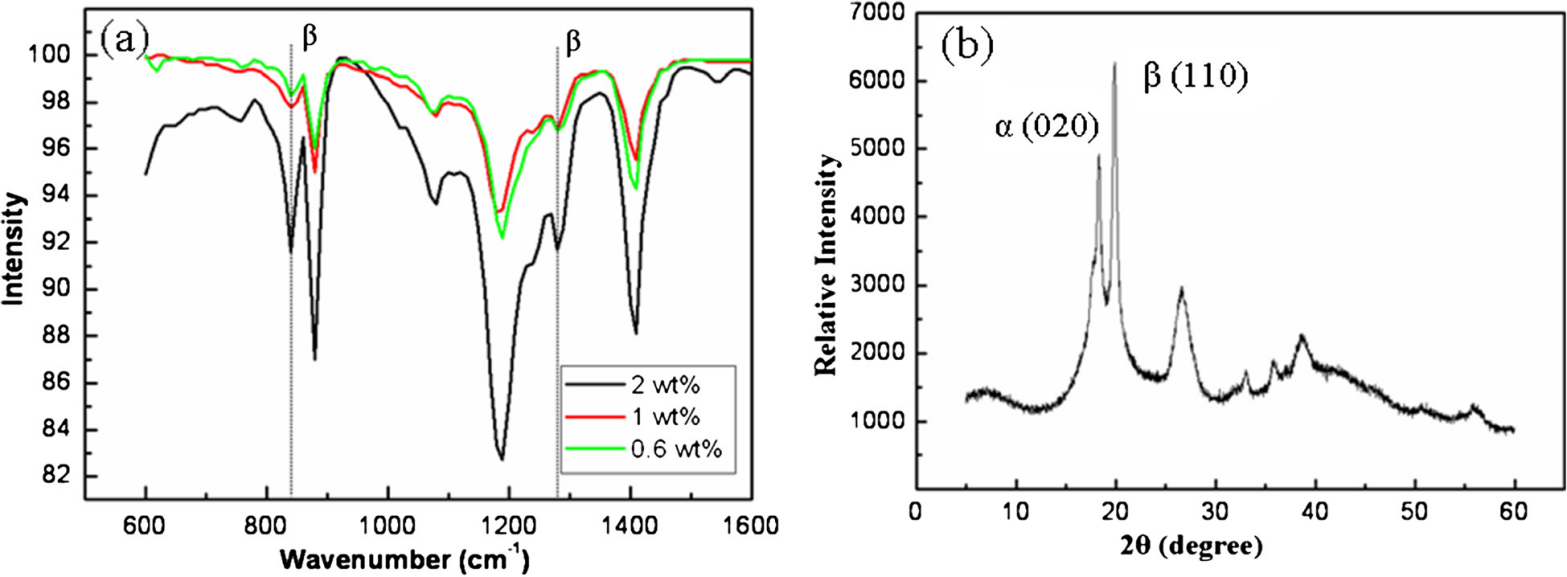
**FTIR spectra and XRD diffraction patterns of the aligned electrospun PVDF/MWCNT fibers: (a) FTIR spectra of some aligned electrospun PVDF/MWCNT fibers with MWCNTs of 0.6, 1, and 2 wt.%. (b)** XRD diffraction patterns of the aligned electrospun PVDF/MWCNT fibers with MWCNTs of 2 wt.%.

As we know, if the rotating speed of the collector is too low, the alignment of fibers is not able to be initialized, while fibers lead to fracture with an extreme rotating velocity [24]. Here the suitable rotating speed of the collector was selected of 150 to 170 rpm, and in this course, all the aforementioned solutions can be electrospun into well-aligned fibrous arrays. Figure 2a,b shows SEM images of the aligned electrospun PVDF/CNT ultrafine fibers with MWCNTs of 2 and 0.6 wt.%, respectively. We can see that the fibers have regular diameters ranging from 200 to 1200 nm and most of them are at the vicinity of 800 nm. However, besides some of the MWCNTs inside the electrospun fibers with well orientation along the fiber axis, most MWCNTs form agglomerates or exhibit curved or wavy conformation rather than straight, as shown in Figure 2c,d. This phenomenon is thought may due to either their inherent dispersion characteristic properties of MWCNTs in polymeric precursor solution [15] or they cannot fully be stretched under the electrospininng parameters (e.g*.*, the applied electric field force). For example, according to a previous work of Dror et al. [25], the MWCNTs are more sensitive to distortions during electrospinning and are thus incorporated into the fibers in an unoriented form compared with single-walled nanotubes (SWCNTs). On the other hand, the modified electrospinning device with rotating collector can fabricate well-aligned micro/nanofibers by combing electric field force and mechanical force. In this case, a lower voltage is required, which may bring a too low applied electric field force accordingly to stretch the MWCNTs. Figure 3a shows the FTIR spectra of aligned electrospun PVDF/MWCNT fibers with MWCNTs of 0.6, 1, and 2 wt.%, respectively; here the enhancement of *β* phase (infrared band at 837 and 1,273 cm^-1^) with increasing MWCNT content can be observed. It is because not only the higher electric field during electrospinning produces a completely different arrangement of the polarity direction of the PVDF fiber, facilitating the growth of the crystalline structure [19], but also of the increasing amounts of the MWCNTs in the composited fibers [15]. The diffraction peak at 2*θ* = 19.9° in the XRD diffraction pattern (Figure 3b) also corresponds to the *β* phase, where the MWCNT content is of 2 wt.%.

### Mechanical properties

**Figure 4:**
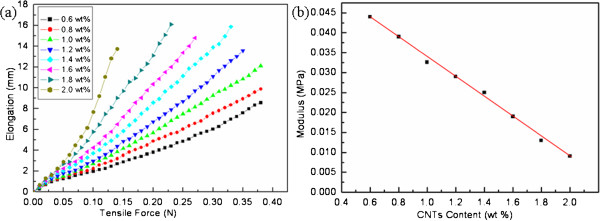
**Mechanical properties of PVDF-MWCNT ultrafine fibers. (a)** Relationship between tensile strength and elongation for composite fibers, containing different CNT contents: 0.6, 0.8, 1, 1.2, 1.4, 1.6, 1.8, and 2 wt.%. **(b)** Relationship between Young's modulus and MWCNT contents in the composite fibers.

Due to a very small dimension, the mechanical characterization of one individual electrospun fiber is still a challenge for the existing test instruments, which is the reason why articles addressing the mechanical tests of individual fibers are rare. Thus, in order to measure the mechanical properties of the as-spun ultrafine fibers, a square area of the membranes (area of 0.01 cm^2^ and thickness of 0.1 mm) based on the aligned electrospun fibers has been tested. Figure 4a shows typical tensile test (along the fibrous axis) results of these composite fibrous mats containing MWCNT contents of 0.6, 0.8, 1, 1.2, 1.4, 1.6, 1.8, and 2 wt.%, respectively. With the same tensile stress, the fibers containing more MWCNTs demonstrated a larger strain, namely, the MWCNTs obviously improved the mechanical properties of the as-spun fibers.

The change in rheological behavior as the concentration of MWCNTs increases, similar to those presented in Figure 4a, has been reported for other polymer/CNT composites and is often called ‘percolation threshold’ [26]. More precisely, one might call the emergence of the static gel network as ‘mechanical percolation threshold’ , to differentiate it from the more traditional electrical percolation [27] or indeed the mathematical problem of percolation of rigid rods [28,29]. In effect, the recording of stress against contents returns the value of Young's (extensional) elastic modulus (*E*). Generally, the relationship between stress and strain of conventional fiber reinforced composite can be expressed as

(1) $$\frac{F}{S}=E\times \lambda$$

Here *F* and *S* are the tensile force and cross-sectional area of fibrous membranes (here is of 0.01 cm^2^ × 0.1 mm), respectively. The stretching ratio, *λ*, can be calculated from

(2) $$\lambda =\frac{x-x_{0}}{x}=\frac{\Delta x}{x}$$

and *x* and *x*_0_ are the lengths of fibrous mat after and before stretching, respectively. Hence, Young's modulus (*E*) can be expressed as

(3) $$E=\frac{F}{S}\times \frac{x}{\Delta x}$$

Figure 4b presents the relationship between Young's modulus and MWCNT contents for the electrospun PVDF/MWCNT fibers. With the content of carbon nanotubes increased from 0.6 to 2 wt.%, Young's modulus is decreased from 4.4 × 10^-2^ to 9.1 × 10^-3^ MPa, e.g*.*, the products' elasticity has been drastically improved with the increasing of MWCNTs.

### Electrical properties

**Figure 5:**
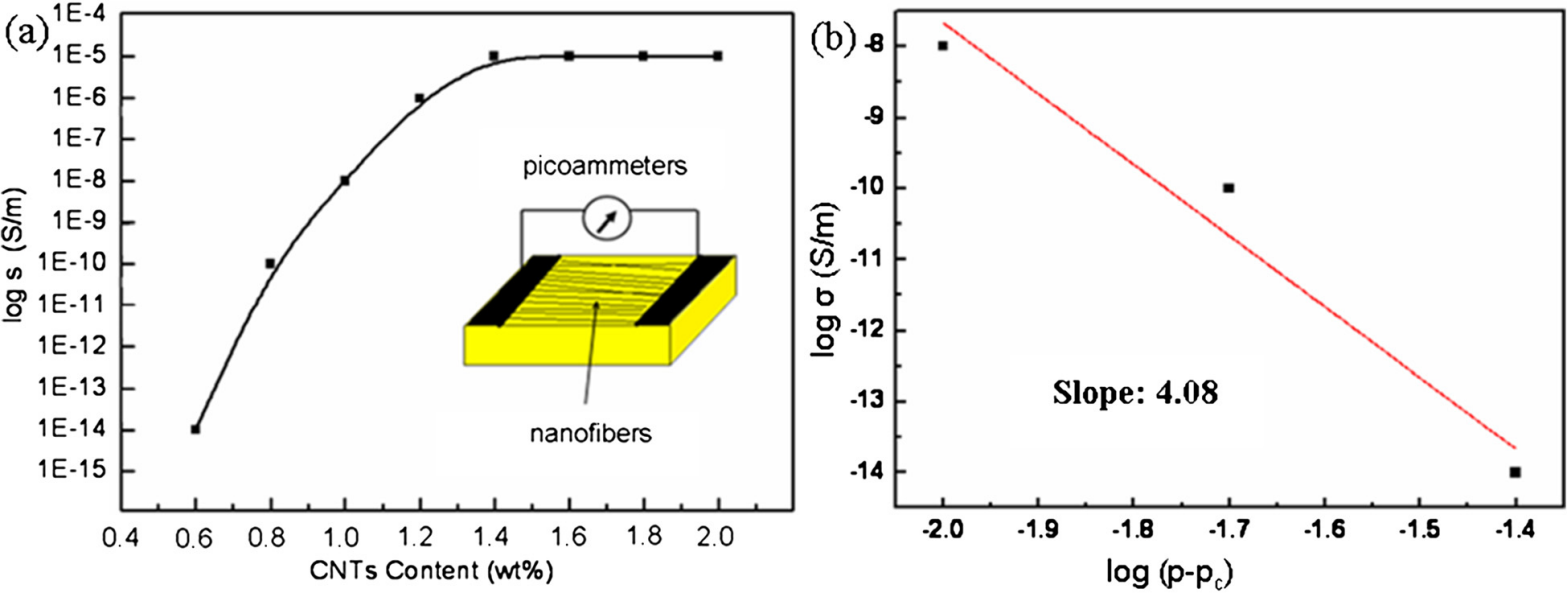
**Electrical properties of PVDF/MWCNT ultrafine fibers. (a)** Relationship between conductivity and MWCNT content for the composite fibers. The inset shows the diagram of electrical measurement*.***(b)***The linear relation of σ* and (*p* - *p*_*c*_) on a log-log plot.

The room-temperature conductivity, *σ* (300 K) of the PVDF/MWCNT fiber membranes, is shown in Figure 5a. The inset is diagram of the experimental sample (area of 0.01 cm^2^ and thickness of 0.1 mm) and electrical measurement. The conductivity of the composited fibrous films increased rapidly with the increasing of MWCNT mass proportion. The room-temperature conductivity of the PVDF/MWCNT fiber membranes with MWCNT content of 0.6 wt.% was 1 × 10^-14^ S cm^-1^. However, when the content of the MWCNTs was 1.2 wt.%, the conductivity changed into 1 × 10^-6^ S cm^-1^, which was eight orders of magnitudes comparing to that of the 0.6 wt.% counterpart. Namely, the presence of MWCNTs significantly improved the conductivity of the polymer fibers due to their highly conductive graphitic structures. The notable increase in conductivity was also owing to the introduction of conducting CNT paths to the PVDF, indicative of percolation behavior, where the filler particles in the polymer matrix are free to move and thereby can form a conducting network at much lower particle concentrations [30].

According to the percolation theory, on the conducting side in the vicinity of the percolation threshold, a universal relationship between the electrical conductivity and the contents of the conductive filler can be described as follows [31]:

(4) $$\sigma \left(p\right)\propto {\left[p-p_{c}\right]}^{t}$$

Here *σ* is the electrical conductivity of the PVDF/MWCNT composited fibers, *p* is MWCNT concentration, *p*_*c*_ is the percolation threshold, and *t* is the critical exponent, which reflects dimensionality of the system and universality class of the problem. Usually, experimental results are fitted by plotting log *σ* vs. log (*p* - *p*_*c*_) and incrementally varying *p*_*c*_ until the best linear fit is obtained [22]. In our experiments, the percolation threshold *p*_*c*_ and critical exponent *t* (the slope of the linear relation of log *σ* to log (*p* - *p*_*c*_)) were of 1.2 and 4.08 wt.%, respectively, as shown in Figure 5b. Besides, it is reported that for the polymer/CNT composites, the exponent *t* is frequently associated with the conducting system dimensionality, namely, with values of *t* ≈ 1.3 (or slightly higher) representing a two-dimensional network while *t* ≈ 2 (or higher) a three-dimensional one [22]. Here the values of *t* = 4.08 indicated a three-dimensional conducting network formed among PVDF/MWCNT composited fibers. As increasing the amounts of MWCNTs in the composites, the density of CNT-CNT junctions increased accordingly, with an enhancive conductivity till the MWCNT content of 1.2 wt.%. After the three-dimensional network had been constructed, the density of CNT-CNT junctions tended to be a constant, therefore, the conductivity of the PVDF/MWCNTs remained stabilized, and the value of log *σ* in Figure 5a was likely to form a platform after the percolation threshold.

## Conclusions

In this paper, the ultrafine fibers of PVDF/MWCNTs were fabricated via a modified electrospinning technique. The mechanical and electrical properties of the as-spun fibers were enhanced evidently by incorporating MWCNTs into the PVDF fibers. With the increase of the MWCNT content, an enhancement of the *β* phase was observed. With the MWCNT mass proportion increased from 0.6 to 2 wt.%, Young's modulus of the composited fibers decreased from 4.4 × 10^-2^ to 9.1 × 10^-3^ MPa. At room temperature, the conductivity of the PVDF/CNT fiber membranes with MWCNT content of 0.6 wt.% was 1 × 10^-14^ S cm^-1^, however, for the 1.2 wt.% loaded, it changed into 1 × 10^-6^ S cm^-1^, and the critical exponent *t* was of 4.08, which proved that a three-dimensional conducting network constructed among PVDF/MWCNT fibers. After the network formed, the density of CNT-CNT junctions tended to a steady value, which led to the conductivity of the PVDF/MWCNT fibers forming a platform after the percolation threshold (MWCNT content of 1.2 wt.%). It is hoped that our results can be helpful for the fabrication of flexible devices, piezoelectric devices, force transducer, etc.

## Competing interests

The authors declare that they have no competing interests.

## Authors’ contributions

SHW and BS designed the experiments. SHW, YW, and BS carried out the electrospinning experiments. LZL tested the mechanical and electrical properties of the samples. WX contributed to the data analysis. All authors read and approved the final manuscript.
